# How do health behaviour interventions take account of social context? A literature trend and co-citation analysis

**DOI:** 10.1177/1363459317695630

**Published:** 2017-03-23

**Authors:** Daniel Holman, Rebecca Lynch, Aaron Reeves

**Affiliations:** University of Sheffield, UK; London School of Hygiene & Tropical Medicine, UK; London School of Economics and Political Science, UK

**Keywords:** bibliometrics, co-citation analysis, health behaviour, health interventions, social context, social determinants, social science

## Abstract

In recent years, health behaviour interventions have received a great deal of attention in both research and policy as a means of encouraging people to lead healthier lives. The emphasis of such interventions has varied over time, in terms of level of intervention (e.g. individual vs community) and drawing on different disciplinary perspectives. Recently, a number of critiques have focused on how health behaviour interventions sometimes sideline issues of social context, framing health as a matter of individual choice and, by implication, a personal responsibility. Part of this criticism is that health behaviour interventions often do not draw on alternative social science understandings of the structured and contextual aspects of behaviour and health. Yet to our knowledge, no study has attempted to empirically assess the extent to which, and in what ways, the health behaviour intervention field has paid attention to social context. In this article, we undertake this task using bibliometric techniques in order to map out the health behaviour intervention field. We find that the number of health behaviour interventions has grown rapidly in recent years, especially since around 2006, and that references to social science disciplines and concepts that foreground issues of social context are rare and, relatively speaking, constitute less of the field post 2006. More quantifiable concepts are used most, and those more close to the complexities of social context are mentioned least. The document co-citation analysis suggests that pre 2006, documents referring to social context were relatively diffuse in the network of key citations, but post 2006 this influence had largely diminished. The journal co-citation analysis shows less disciplinary overlap post 2006. At present, health behaviour interventions are continuing to focus on individualised approaches drawn from behavioural psychology and behavioural economics. Our findings lend empirical support to a number of recent papers that suggest more interdisciplinary collaboration is needed to advance the field.

## Introduction

Health behaviour interventions (HBIs) are currently highly topical in public health and health policy (National Institute for Health and Care Excellence (NICE), 2007, 2014). Individual behaviour is commonly seen as the primary driver of the biggest health challenges facing high-income countries, such as obesity and diabetes, and so attention has turned to how people can be encouraged to lead healthier lives (Bambra et al., 2011; Goldberg, 2012; Katikireddi et al., 2013; McCartney et al., 2013; Popay et al., 2010). However, as far back as John Snow, epidemiologists (and more recently social scientists) have demonstrated that health is determined by social structure as well as individual choice (Brady and Collier, 2010), from the level of wide-scale political and economic climates, to local communities, social networks and practices of everyday life. There is an extensive literature on the social determinants of health that illuminates its causes at multiple levels. However, a number of papers have argued that this literature does not inform the design and evaluation of HBIs and thus may be undermining the effectiveness of these interventions (Blue et al., 2016; Cohn, 2014; Glanz and Bishop, 2010; Glass and McAtee, 2006; Thorlindsson, 2011). Yet to our knowledge, no study has attempted to empirically assess the extent to which, and in what ways, the HBI field has so far incorporated the wider social context of health in the design and evaluation of HBIs. In this article, we undertake this task using bibliometric techniques in order to map out the HBI field, analyse the extent to which social context has been represented within it and evaluate how this has changed over time. We do not restrict our analysis by country, but focus our review of the policy context on the United Kingdom given our familiarity with it, illustrating how the United Kingdom reflects the broader field.

### Historical context and current approaches

Public health interventions have placed differential emphasis on the multiple levels (e.g. individual, community and national) of the determinants of health over time (Glanz and Bishop, 2010). One of the earliest and most important individual-level interventions was the Multiple Risk Factor Intervention Trial (MRFIT), conducted in the 1970s. The trial had high expectations, but failed to deliver significant change. This was interpreted as evidence of the importance of community-level factors on health, which led to a series of interventions intended to alter the context in which people made health-related choices (Cutler, 2004). But these community-wide interventions also had very modest effects and prompted a return to more individual-level analysis, now drawing on psychology and behavioural economics to design interventions grounded in theories of behaviour change (Cutler, 2004). Of course, the community- and national-level determinants of health were not forgotten, and a vast literature has examined how these factors affect health using observational studies (CSDH, 2008). Through this work, there are signs of a renewed focus in public health on wider determinants of health (Glanz and Bishop, 2010). Indeed, this shift is also reflected in recent policy documents. In the United Kingdom, guidance from both NICE (2007, 2014) and the Medical Research Council (MRC, 2008, 2014) has highlighted the importance of cultural acceptability, socio-economic position and the ‘social, political or geographical context in which interventions take place’ (MRC, 2008: 6).

One reason for the differential emphasis on the social in HBIs is that the central theories of action and interaction that have informed these interventions have been grounded in disciplines that primarily attend to individual behaviour. Psychology, for example, is concerned with intra-individual concepts such as cognitions, beliefs, knowledge, perceptions and motivations. Likewise, behavioural economics is concerned with understanding social behaviour in terms of rational choice and utility maximisation, and so largely views aggregate patterns as the product of individual-level processes. Therefore, HBIs which emerged largely from psychology and economics have also tended to focus on behaviour change and individual-level interventions rather than determinants located in social groups, communities, cultures and nations.

Of course there are different approaches within these broad disciplinary orientations, and these disciplines do not deny ‘the social’ nor ignore the importance of the wider determinants of health. Some psychological techniques, such as motivational interviewing, acknowledge participants’ interpersonal circumstances and thereby try to incorporate social context into their analyses,^1^ and economists such as Angus Deaton – the recent Noble Prize winner – have also been cautious about the value of experimental approaches to health-related questions, arguing that these interventions often fail to estimate quantities of interest to policymaking and that experiments do not constitute a ‘gold standard’ of evidence because they lack generalisability (Deaton, 2014). While public health as a whole is very much aware of the influence of public policy and social context on health (Braveman et al., 2011), approaches to behaviour from health psychology and behavioural economics largely frame how the social world should be understood via its relation to the individual and as something impacting, and therefore knowable through, individual understandings, constraints and behaviours. This approach differs from other disciplines where the social is something beyond this individual-level filtering. Disciplines such as sociology and anthropology are explicitly concerned with ‘society’, which they view as more than just a collection of individuals. They might focus on how societies are structured, for example, according to class and gender, and how these social structures and other social and cultural dynamics influence (and are influenced by) the circumstances in which people live. Implicit in this approach is a concern with social interactions and relationships, which Pescosolido (2007) argues is the defining feature of sociology.

When it comes to developing and considering the impact of HBIs, these two different approaches to the social world suggest different methodologies. For the former disciplines, the social can be seen as akin to a variable which can be understood through looking at individuals and individual-level interventions. For the latter, interventions, and methods to evaluate effectiveness, should take account of the relational aspects between people and their surroundings. Such disciplinary differences therefore generate different *kinds* of interventions and methodologies as they suggest different ways in which HBIs might ‘work’ and how their impact might be measured. These differences become important if we consider the ongoing concerns that the current approach to HBIs has had limited effectiveness and may be improved.

At present, HBIs consistently report a small, but significant effect (Ashford et al., 2010; Kamath et al., 2008; Williams and French, 2011). Like with the MRFIT programme, such results are often unsatisfying and so studies often call for further research, for example, including longer trials and more rigour in defining and measuring target behaviours (Kamath et al., 2008). In contrast, some prominent thinkers in the field are calling for redirecting efforts at researching real-world effectiveness (Greenhalgh, 2012; Kessler and Glasgow, 2011), and ‘pragmatic trials’ have attempted to address this issue (Patsopoulos, 2011). It is possible that the small effect often found is underestimated (Deaton, 2014); evaluations of HBIs tend to focus at the individual level and thus are likely to understate the population effect through violating the stable unit treatment value assumption that the treatment of individuals does not affect the outcomes of those in the control group (Morgan and Winship, 2014). Conversely, recent work has shown how social behaviour connects individuals, travelling across social networks (Christakis and Fowler, 2007), and emerges from relationships between those delivering interventions and the target population. Incorporating these insights into HBI evaluations may help explain intervention effects, and a way of doing this may be to open up the ways in which social context is considered in this field.

### Health behaviour interventions and social context

How might HBIs benefit from a greater focus on social context? The social world is complex, resource-limited and ‘messy’, and accounting for this is difficult but important – not least because intervention participants will inevitably come from a variety of circumstances and situations (Glass and McAtee, 2006; McKinlay and Marceau, 2000). HBIs are generally designed so that they can be evaluated through randomised controlled trials which favour uniformity and measurability, and the ‘messier’ parts of the social world are harder to locate through these methods. Some interventions do find ways to take the more complex aspects of the social and cultural context of behaviours into account, and there is some indication that those which do so are more successful (Glanz and Bishop, 2010). One recent promising intervention, *Football Fans in Training*, led by investigators with sociology backgrounds in an interdisciplinary team, targeted men’s obesity at football clubs, giving the intervention the draw of an acceptable and valued delivery setting (Bunn et al., 2016; Hunt et al., 2014). The intervention had positive outcomes (Hunt et al., 2014) and is now being extended across Europe. Similarly, another recent study used work on social networks and obesity to target interventions to certain network members to successfully increase uptake of multivitamins across the whole community (Kim et al., 2015).

To examine the impact of interventions, some social scientists have recently turned their attention to practice theory (Blue et al., 2016; Maller, 2015; Veenstra and Burnett, 2014) which attempts to move beyond more individualised notions of ‘attitudes, behaviour and choices’ by, for example, considering eating as a social phenomenon, comprising meanings (e.g. cultural conventions), materials (e.g. tools) and tacit and explicit competences (e.g. embodied skills) (Maller, 2015). This framework allows for acknowledging the habitual dimensions of interaction shaped by culture, which may be missed by stressing internal psychological processes. Although practice theory has taken off in some fields such as environmental sciences (Hargreaves, 2011), it remains to be seen whether it will do so in public health, and the HBI field in particular.

This debate on the limitations of the individualistic nature of HBIs suggests that issues of social context are again coming to the fore in efforts to understand health behaviour. However, to date, these debates have lacked empirical investigation to document the extent to which social context has been and continues to be used in the HBI field. In this article, we seek to contribute to the debate using bibliometric analysis, first by analysing trends in the literature and then using co-citation analysis to map out how the HBI field has taken account of social context and how this may have changed over time.

## Methods

In contrast to common methods of review in public health (e.g. systematic reviews), bibliometric analysis is (typically) not concerned with examining the content of papers to summarise what is known on a particular issue, but rather with mapping out the scientific field by analysing its literature to uncover patterns, trends and relationships. Our aim in doing this is to explore which disciplines and concepts dominate the field, with particular attention paid to those concepts which relate to social context and HBIs. We have taken a broad approach to defining the field, explicitly searching across differing conceptions of social context within diverse methodological approaches and disciplines.

We used two bibliometric methods to do this, each with a number of stages. First, we carried out a literature trend analysis which involves searching documents’ key fields (in our case, cited documents, author affiliation, abstract, key words and journal title) for concepts to detect ‘delineating subject areas, growing subfields, or disciplinary patterns’ (De Bellis, 2009). Previous examples of papers using this method can be found by Injuk and Van Grieken (2003), De Bakker et al. (2005) and Ha et al. (2009). We then searched for, and then iteratively refined, terms we identified as signifiers of social context based both on this literature review of papers and drawn from our own experiences as researchers in this field. This search produced terms relating to methods and concepts, as well as disciplines present in this field which linked to these. Psychology, sociology, anthropology, epidemiology, economics, health economics and geography were therefore added as search terms, as were ‘multilevel’ and ‘hierarchical’ as signifying acknowledgement of the multilevel determinants of health. Conceptual terms searched for were as follows: culture, income, social determinant, ethnic, gender, disparity, peer, social support, ecologic, norms, inequality, poverty, social network, socio-economic/socioeconomic and social environment. While these are clearly not an exhaustive list of signifiers of social context in the HBI field, these key terms represent a range of social contextual issues, which, crucially for our purposes, allowed us to track trends over time. Some initially identified terms gave too few results to graph (social context, values, upbringing, inequity, resources, social capital, social structure and capital), were too general or had too much overlap with other usage to be useful (sex, community, situation, status and qualitative). Wildcards were used so that different forms of the words, for example, inequality/inequalities, were picked up.

Second, we then undertook a co-citation analysis of the HBI literature. This method is based on the assumption that documents cited together share some kind of intellectual affinity. Documents identified as co-cited are situated within a network map showing how these, and therefore the ideas within them, sit in relation to each other across the field. As De Bellis (2009) notes, ‘co-citation analysis may be used to trace the map of relationships among documents/key concepts, to outline and graphically visualize the structure of a research field, its connections with other fields, and its articulation into subfields and new research fronts’ (p. xxvi). Importantly, for the longitudinal aspect of our analysis, ‘co-citation patterns change as the interests and intellectual patterns of the field change’ (Small, 1973). To produce such maps, bibliometric software (in our case BibExcel;^2^ Persson et al., 2009) is used to extract the cited references from an identified corpus of documents (see below) which are then sorted by number of citations. Commonly cited documents are linked in relation to the number of times they are cited together in other documents. In our analysis, we selected the 100 most cited documents for each time period, allowing for a good balance of breadth and depth. We then fed these data into a tool designed to help visualise bibliometric networks – VOSviewer.^3^ Recent examples of co-citation analyses and this method can be found in Stuckler et al. (2015) and Callard et al. (2013), and details on the algorithm the software uses to visualise the clusters can be found in Eck et al. (2009).

**Figure 1.**
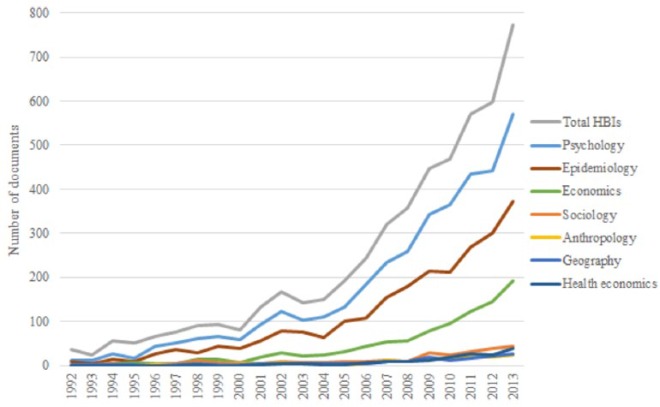
Disciplinary representation in HBIs. Graph lines represent the proportion of documents that mention different disciplines.

Since we were concerned with changes over time, the co-citation analysis was split into two maps: pre and post 2006, this date being approximately when the number of HBI papers started to increase substantially (see Figure 1). As our explicit concern was to map the influence of social context in the HBI field, we coded 200 documents (100 for each time period) identified in the co-citation analysis according to whether their abstracts referred to social context, for example, by acknowledging the heterogeneity of social groups or the social world in the design, implementation or analysis of interventions (see Online Appendix 2). This process was carried out independently by D.H. and A.R., and interrater reliability was good, at 86 per cent agreement (0.59 Cohen’s kappa) representing moderate to substantial agreement (McHugh, 2012). The two coders then discussed disagreements in order to draw out the logic of the coding process so that papers were coded according to consistent criteria. Finally, we carried out a journal co-citation analysis to map out the key journals in the field, their relationship with each other and how this changes pre and post 2006. The method is identical to the earlier document co-citation analysis, but journals, rather than documents, are the units of analysis.

Bibliometric methods require a corpus of literature that represents the field of interest as the data source. The HBI field primarily comprises papers that report on interventions but also includes other documents, for example, books on behaviour change techniques, statistical papers and evaluations. Thus, our search strategy was deliberately broad to capture a representation of the field. We used Scopus for the search instead of Web of Science for its better health coverage (Li et al., 2010). The following search strategy was used (see Online Appendix 1 for full search terms):

- The title included ‘intervention’ or ‘trial’ and excluded ‘meta analy*’ or ‘review’ (to avoid duplication).
- ‘Health’ and ‘behav*’ appeared within five words of each other in the title, abstract or keywords (this excluded many non-HBIs/trials).
- The subject was either health sciences or social science and humanities.
- Literature was searched from 1980 as this was approximately when interest in ‘lifestyle’ behaviours proliferated (Petersen and Lupton, 1996).
- The sources searched were English language journal articles only.

This resulted in a corpus of 5230 documents published from 1980 to 2013. This method was effective at identifying literature of the HBI field on face validity. An important point to note here is that our search strategy would have only picked up papers that explicitly referred to health behaviour and that mentioned interventions or trials in the title. Some community-level interventions that may have had an effect on health behaviour but were not focused on it or were not framed as interventions may have been missed. Nonetheless, our strategy reasonably identified a corpus of papers focused on the topic of changing health behaviour in line with the substantive topic of this article.

## Results

### Literature trend analysis

Figure 1 shows that overall the number of HBI documents has increased in recent years, especially since around 2006.^4^ As the number of HBIs published in peer-reviewed journals has increased, so has the number of HBIs citing the psychological literature. References to disciplines more associated with understandings of social context, such as sociology and anthropology, are minimal pre 2006 and have not followed the increase in HBIs post 2006, in effect showing a peeling away of these disciplines from HBI research. In other words, as HBI research continues to grow, references to these disciplines constitute a smaller proportion of all documents cited. In contrast, as the number of documents has increased, there has been a rise in the number of documents citing the epidemiology and economics literature, although ‘health economics’ as a specific search term remains a small sub-set of these citations. Of the pre-2006 documents, 56.2 per cent were from the United States and 10.3 per cent from the United Kingdom. Post 2006, these figures were 50.6 per cent and 14.2 per cent. We then substituted the term behaviour for promotion to see whether the increase was due to interventions being referred to using different terminologies over time. This was not the case, as the latter term showed an almost identical graph. We also carried out a search using some completely unrelated terms (bridge, acoustics, metal) as well as the term ‘public health’ to see whether the increase around 2006 was due to some kind of artefact, but this was not the case.

**Figure 2.**
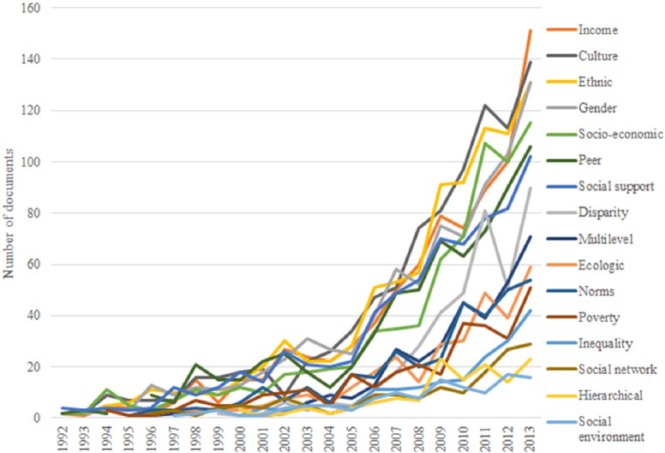
Representation of social context in HBIs. Graph lines represent the proportion of documents that mention different concepts.

Figure 2 traces the number of articles included in our literature search that mention concepts relating to social context. The first point to note is that only a small proportion of documents mention such concepts. Using Figure 1 for comparison, in the most recent year 2013, the highest proportion of documents which have referenced any one concept is 19.5 per cent (for the term income). The graph also shows that more quantifiable concepts such as income, ethnicity, gender and socio-economic were mentioned more regularly than concepts related more closely to the complexities of social context or inequalities such as poverty, social network, norms or social environment. The former set of quantifiable concepts has followed the increase in the total number of documents to a greater extent than the latter set of terms, somewhat mirroring the disciplinary trend search. Apart from these overall trends, there were no obvious patterns with respect to whether certain concepts became more or less popular over time.

### Document co-citation analysis

We then proceeded with the document co-citation analysis in order to map out the key authors in the field and where their ideas sit in relation to each other. The most highly cited documents, authors and journals are shown in Table 1. The influence of health behaviour theories is clear; documents relating to these theories are by far the most highly cited in the field. Their American origin is reflected in where the most highly cited journals are based, although it is worthy of note that *BMJ* and *The Lancet* are highly cited. In addition to being UK-based, these journals give substantial coverage to the social aspects of medicine compared with many other medical journals.

**Table 1.** Top 10 cited documents, authors and journals.

Cited document	Frequency	Cited author	Frequency	Cited journal	Frequency
Bandura A (1986) Social foundations of thought and action	145	Bandura A	795	*JAMA*	1634
Bandura A (1997) Self-efficacy	74	Prochaska JO	538	*American Journal of Public Health*	1327
Ajzen I (1991) The theory of planned behaviour	66	Sallis JF	445	*BMJ*	1139
Prochaska JO et al. (1992) In search of how people change	51	Diclemente, CC	409	*American Journal of Preventive Medicine*	978
Prochaska JO and Diclemente CC (1983) Stages and processes of self-change in smoking	45	Glasgow RE	372	*Preventive Medicine*	965
Baron RM and Kenny DA (1986) The moderator-mediator variable distinction in social psychological research	43	Miller WR	348	*Journal of Consulting and Clinical Psychology*	958
Cohen J (1992) A power primer	41	Cohen J	344	*Health Psychology*	915
Bandura A (1977) Self-efficacy	38	Ajzen I	343	*The Lancet*	854
Ajzen I and Fishbein M (1980) Understanding attitudes and predicting social behaviour	34	Rollnick S	341	*Health Education Research*	657
Radloff LS (1977) The CES-D Scale	31	Brug J	288	*Pediatrics*	653

**Figure 3.**
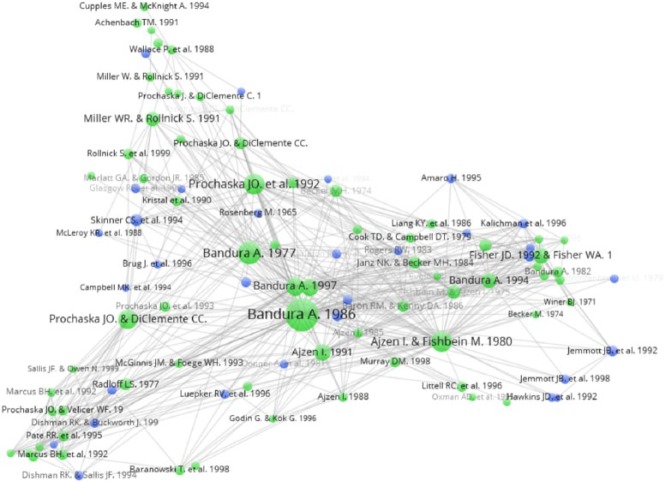
Document co-citation analysis for pre-2006 HBI papers. Blue circles represent documents that refer to social context; green circles are all other documents.

For the document co-citation analysis, we split the analysis pre and post 2006 given the increase in the number of HBIs around this time (see Online Appendix 2 for list of documents). The pre-2006 analysis is given in Figure 3. The centrality of Bandura’s concept of self-efficacy is clear in the pre-2006 citation analysis. As indicated by the size of the nodes, Bandura’s 1977, 1997 and 1986 documents on the concept were the most highly cited and central in the network. Other popular documents were Prochaska et al. (1992), Prochaska and DiClemente (1986) and Ajzen and Fishbein (1980). The network also shows a number of distinct camps. Documents in the bottom left of the network focus on physical activity and exercise, those towards the right on sexual health and those at the top are slightly more disparate, covering child behaviour, alcohol use and motivational interviewing. The documents which referred to social context were dispersed throughout the field, suggesting these ideas fed into various types of HBIs but not necessarily in a systematic way and there does not appear to be any non-psychology citation classics among these HBIs. Two of the main types of social context documents were community studies and interventions targeted at disadvantaged populations.

**Figure 4.**
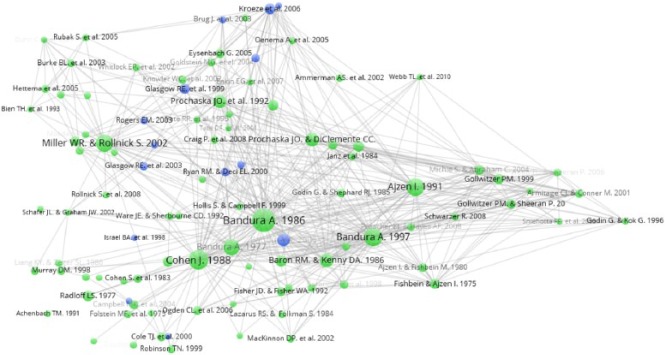
Document co-citation analysis for post-2006 HBI papers. Blue circles represent documents that refer to social context; green circles are all other documents.

The post-2006 field of documents is more dispersed than the pre-2006 field, with less easily identifiable scholarly camps (Figure 4). Some of this dispersion might have been expected to occur as a function of the increased number of citations. However, documents that referred to social context have become fewer in number (from 31 to 14), and these have to some extent clustered in the space of ideas. With one or two exceptions, these documents were not present in the pre-2006 analysis and no longer cover community interventions or interventions targeted at disadvantaged populations. The group of documents at the top of the diagram are on tailoring, with a focus on Internet-based HBIs. In a sense, these documents have become a specialism in the field. Bandura’s ideas have, however, remained central and arguably have been ‘canonised’. Two additional central documents here which were not in the previous diagram are Cohen (1988) and Baron and Kenny (1986), which are both statistical methods papers. The bottom right of the diagram contains virtually no documents referring to social context. These documents centre around the theory of planned behaviour (Godin and Kok, 1996), belief, attitude, intention and behaviour (Fishbein and Ajzen, 1975) and statistical techniques (MacKinnon et al., 2002).

### Journal co-citation analysis

**Figure 5.**
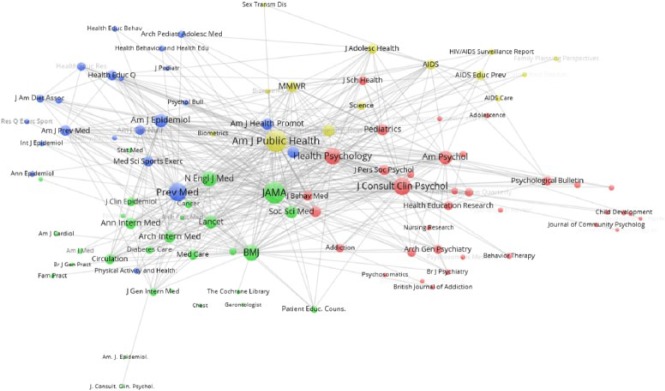
Journal co-citation analysis for pre-2006 HBI papers. See text for description of nodes.

Next we examined the patterns of co-citation analysis between journals to capture any disciplinary silos. Again, we split the analysis between those documents published before and after 2006. The pre-2006 journal co-citation analysis (Figure 5) suggests that there were four distinct clusters, which broadly represent different subject areas. The green and red clusters are straightforward and represent medical and psychology journals, respectively. The blue cluster is more mixed, but relates mostly to health promotion, covering health education, preventive medicine and diet/exercise. The yellow contained scientific and statistical journals, though also included the *American Journal of Public Health*, and a number of AIDS/HIV journals. Although each cluster has a number of peripheral journals, there is a fair degree of overlap between the clusters, with the centre space of the diagram where the most highly co-cited journals are present being shared fairly equally between the different clusters. *Social Science and Medicine* was the most popular journal with a specific social science focus. Given its importance to the representation of social science in the field, it is interesting to note that it occupies a central position in the diagram and serves to bridge the medical and psychology clusters. Yet, despite its centrality, none of the papers from this journal are included in the 100 most cited documents (the highest cited *Social Science and Medicine* paper with three citations is ‘Making sense of randomization; responses of parents of critically ill babies to random allocation of treatment in a clinical trial’ (Snowdon et al., 1997)).

**Figure 6.**
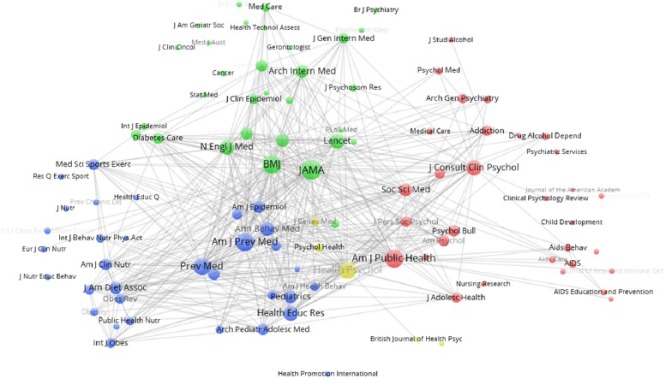
Journal co-citation analysis for post-2006 HBI papers. See text for description of nodes.

Post 2006, the clustering algorithm identified a set of clusters that have less overlap, suggesting more journal self-referencing in recent years (Figure 6). Broadly, three main clusters were identified. Disciplinary boundaries are less obvious than pre 2006. While the green cluster still represents more medical-focused journals and red psychology journals, some psychology journals are now identified in the smaller yellow cluster. The blue health promotion cluster is now more dominant and seems to include a separate sub-cluster of journals focused specifically on diet and exercise, reflecting common targets of recent HBIs. Some of the journals have now been identified by the clustering algorithm as belonging to different clusters, and it is particularly interesting to note that *Social Science and Medicine* has now been identified among the psychology journals, moving away from the centre. The psychology cluster has also now incorporated the *American Journal of Public Health*, alongside various AIDS/HIV journals.

## Discussion

In this article, we observed a general trend of a ‘peeling away’ of social context from the HBI field; the volume of outputs has increased substantially in recent years, but the representation of social science disciplines and concepts centred around social context has not followed suit. This trend must be seen in the wider context of academic publication, social science and politics of research.

Some of the general trend we observe almost certainly reflects the increased volume of outputs across all disciplines, linked with the academic obsession over metrics and rankings (Gruber, 2014) – although we did attempt to control for this using comparator search terms, which suggested that the increase was over and above that for other fields. The obsession with metrics is also a likely driver for the increasing disciplinary segregation we observed in the post-2006 field: as noted by Ioannidis (2015), impact factors can be ‘gamed’ through self-citation (including direct, co-author, collaborative and coercive self-citation). This would likely result in disciplinary self-citation. Our findings here are consistent with psychology having the highest rate of self-citation compared with other social sciences (CWTS, 2007; categorised as a social science here for sake of comparison).

Social science disciplines and their theories evolve over time in terms of scope and focus. Haney (2008) argues that the social sciences have become increasingly Americanised and characterised by the use of quantifiable and psychological concepts. Similarly, it has been argued that psychology has tended to align itself more with medicine and other science, technology, engineering and mathematics (STEM) disciplines (Gergen, 2001; Petocz, 2011). Goldberg (2012) explicitly links the American emphasis on individual rights and responsibilities with methodological individualism in health promotion. Even theories within disciplines change focus over time. For example, Bandura’s (1986) dominant social cognitive theory originally sought to understand the interplay of ‘cognitive, behavioral, and environment factors’, including a consideration of social-network influences and social change. Yet in practice, our findings suggest that these more social elements of the theory are downplayed.

These shifting emphases are a reminder that research fields are based within a wider political context. While there is a danger in invoking neoliberalism to explain almost any health phenomenon (Bell and Green, 2016), it seems particularly apt to describe the growing trend in individual-focused HBIs in relation to neo-liberal discourse around individual responsibility for health and behaviour (Blue et al., 2016; Cohn, 2014; Goldberg, 2012; Thorlindsson, 2011). Through such a lens, it is individual ‘health behaviour’ which is the key site of risk and blame, and the responsibility lies with the individual, rather than governments, to manage health. As Glass and McAtee (2006) state, much of the literature ‘continues to treat behaviours such as diet, smoking, violence, drug use, and sex work as if they were voluntary decisions, without regard to social constraints, inducements, or pressures’ (p. 1652). Similarly, Baum and Fisher (2014) argue that ‘the individualism of neoliberal theory offers little space to support a view that health is primarily created by the structure which powerfully shapes peoples’ lives, including the dominant economic structure’ (p. 61).

This discourse can be seen in key policy documents often cited in research bids, such as the Ottawa Charter in the United States (WHO, 1986), the *The Wanless Review* (Wanless, 2002) and *Choosing Health* white paper (Department of Health, 2004) in the United Kingdom. This might help explain the well-recognised phenomena of ‘lifestyle drift’, whereby research and policy recognise the need for upstream social or policy interventions only to revert back to individual-focused downstream interventions (Marmot et al., 2010; Popay et al., 2010), which are those most likely to increase inequalities (Lorenc et al., 2012). With respect to the government-commissioned reports on health inequalities (The 1980 Black Report, 1998 Acheson Enquiry and 2010 Marmot Review), Bambra et al. (2011) make the point that the wealth of evidence on health inequalities that has accumulated – although tending more towards description than prescription of what can be done – has for the most part not informed policy efforts. What is needed is a focus on suggestions for how to solve inequalities and creating a climate where tackling the issue is seen as publicly and politically desirable.

With this context in mind, our findings suggest a number of potentially useful avenues for future research and policy. In terms of the sheer volume of output, it is evident that the HBI field is a burgeoning one that has more and more funding directed towards it. Indeed in the US context, from 2010 to 2012, the National Institute of Health has spent US$2.2–US$2.6 billion on behavioural interventions (Calitz et al., 2015). In times of austerity, the field must be scrutinised to see how it compares in terms of cost-effectiveness with other more upstream-focused areas of health research and policy; researchers, academics, policy-makers, clinicians and other stakeholders have a role to play here. Crucially, however, this is unlikely to happen if the field becomes the exclusive province of a narrow range of disciplines that tend not to be concerned with critical reflection or wider contextual issues. In other fields – ageing being a prime example – it is increasingly acknowledged that the only way to solve complex societal challenges is through interdisciplinary working. A growing number of voices within the health behaviour field itself (e.g. Diehl et al., 2016; Katikireddi et al., 2013; Kaufman et al., 2014; Stetson et al., 2016) are echoing this sentiment. Of course one would expect disciplines such as psychology and medicine to remain central given the methodological and conceptual nature of HBIs; however, there is space also for incorporating more nuanced understandings of the social context in which HBIs take place. One strong impediment here is the length of funding cycles (e.g. 5 years typically for US National Institutes of Health (NIH) grants) which lend themselves to individual-focused interventions, a point also made in the Foresight report on obesity (Butland et al., 2007). Importantly, the different disciplines involved should not only be present as a token gesture or for intervention evaluation but also be fully implicated in design, implementation and analysis.

Our findings regarding the use of concepts over time suggest that in its early development the HBI field tended to draw upon more nuanced and complex concepts relating to social context, such as poverty, norms or environment, whereas recently this has given way to the use of more individualised and quantifiable concepts. This shift is reflected in the co-citation analysis which showed that key papers incorporating issues of social context changed focus from being typically on community studies or studies targeting minority populations, to computer-based tailoring, especially according to illness characteristics. Thus, these earlier social context publications have failed to cement their influence, in contrast to the canonisation of others, for example, by Bandura and Prochaska. That the term ‘culture’ was mentioned most frequently out of all concepts (over all years) suggests that this is a concept the field is receptive to and may serve as an interdisciplinary common ground. As noted, its importance has been acknowledged by *The Lancet* and the ‘Health 2020’ framework (Fietje and Stein, 2015; Napier et al., 2014). While the latest MRC guidance pays scant attention to culture, it is promising that NICE’s Behaviour Change [PH6] guidance makes numerous references, for example, stating that interventions should be planned with individuals, communities, organisations and populations and ‘take account of the circumstances in which people live, especially the socioeconomic and cultural context’ (NICE, 2007). Our evaluation of the literature suggests that a new guidance document specifically focused on culture, and authored by the MRC or NICE, would have the best chance of occupying a central place in the ‘network of ideas’ as shown in Figure 4. A pre-existing model such as that by Sorensen et al. (2003) would be a sound starting point. The complexity of culture represents a difficult challenge for such a document – see, for example, Kok et al. (2012) and Okwaro et al. (2015) on the complexity of local and political context, Bonell et al. (2012) on the implications of complexity for evaluation and Asad and Kay (2015) on the multidimensional nature of culture. New guidance could also suggest how research teams should be balanced in terms of disciplinary representation.

Ultimately, without acknowledging the reality of the wider social and cultural determinants of health, HBIs will be of questionable long-term effectiveness. Our analysis suggests that the following might be useful avenues to explore in moving the field forward:

- The growth of the HBI field calls for much more critical reflection over its direction and cost-effectiveness. Researchers, policy-makers, academics, clinicians and other stakeholders have a role to play.
- Interdisciplinary engagement especially involving disciplines concerned with critical reflection and contextual issues should be pursued for a number of reasons, the most fundamental of which is that behaviour, although manifest at the individual level, is deeply contextual.
- A range of disciplines should be involved in all stages of HBI research, including design, implementation and evaluation.
- A focus on culture seems one promising way forward as it represents a common ground between different disciplines, notwithstanding further work to be done to unravel its complexity.
- Key policy actors (such as the MRC and NICE in the UK context) should further promote the role of social and cultural context in HBIs, which could include guidelines for interdisciplinarity.

Interestingly, Glass and McAtee (2006) argued in 2006 that behavioural science was at a crossroads in public health. The analysis here suggests that the road taken has been one of increased individualism and downstream interventions. Yet there are signs that researchers and policy-makers might be beginning to pay more attention to the role of social science and social context. The influence this has on the HBI field remains to be seen.

## Limitations

Bibliometric analysis represents a ‘broad brush’ approach, first, in the sense that documents are not reviewed in detail to ensure they meet preset criteria, and, second, in the case of the literature trend search, the analysis concentrates on mentions of terms in citation records rather than on the full text. It is possible that social science conceptualisations feed into the field in more subtle and nuanced ways. Nonetheless, the method was effective in identifying historical trends. In addition, searching abstracts identifies either what authors view as important in summarising their documents or what journal editors are looking for; both of these are good indicators of the state of the field. Another limitation is that the majority of documents were from the United States, which has a different, although we would argue highly related, research and policy context than the United Kingdom. The document co-citation analysis was limited in terms of selecting the ‘top’ cited documents, while it might be that the majority of social science documents were less influential than this and were missed in the analysis. However, the identified trends over time and comparison with the first stage of the analysis suggest this was not the case. Similarly, we did not search the grey literature, although it may well have been that the representation of social science was more prominent here.

## Conclusion

This article has explored the nature and extent of the representation of social science disciplines and concepts relating to social context in published literature on HBIs. It has found that such concepts are rarely represented in the HBI field, and when they are, individual and quantifiable concepts are used most. Over time, concepts relating to social context have constituted less of the field, especially since 2006 when there was a particular increase in the number of HBIs. Thus, the overall trend has been towards the further individualising of health behaviour, despite recent research and policy attention on interdisciplinary working and the need to pay attention to social and cultural factors. While there were a number of social context documents feeding into various areas of the field pre 2006, after 2006 this influence waned, and the only documents which referred to social context in some way were those centred on computerised tailoring. Some recent work in both research and policy is beginning to explore the social complexity around interventions and the promise of interdisciplinary perspectives. Focusing efforts around the importance of culture is one likely way in which such work can proceed.

## Supplementary Material

Supplementary material

Supplementary material

## Notes

1. One of the originators of motivational interviewing has since argued that it requires broadening to incorporate a fuller array of social context concerns (Emmons and Rollnick, 2001).

2.
http://homepage.univie.ac.at/juan.gorraiz/bibexcel/index.html

3.
http://www.vosviewer.com

4. To aid presentation, the graph shows trends from 1992 onwards as this was when the number of papers started to increase from a nominal level.
