# Supplementary material for: How do health behaviour interventions take account of social context? A literature trend and co-citation analysis
Source: Health (London). 2017 Mar 23;22(4):389–410. doi: 10.1177/1363459317695630 (PMC6377061; doi:10.1177/1363459317695630)
Supplement: Supplementary material [file Web_Appendix_1.pdf]

Web appendix 1 Search Terms

SCOPUS search term to identify all papers

((TITLE(intervention) OR TITLE(trial) AND TITLE-ABS-KEY(health w/5 behav*) AND NOT TITLE (meta-analy*) AND NOT TITLE (meta analy*) AND NOT TITLE(review)) AND SUBJAREA(MULT OR MEDI OR NURS OR VETE OR DENT OR HEAL OR MULT OR ARTS OR BUSI OR DECI OR ECON OR PSYC OR SOCI) AND PUBYEAR > 1979 AND PUBYEAR < 2014 AND (EXCLUDE(SUBJAREA,"BIOC") OR EXCLUDE(SUBJAREA,"NEUR") OR EXCLUDE(SUBJAREA,"PHAR") OR EXCLUDE(SUBJAREA,"AGRI") OR EXCLUDE(SUBJAREA,"IMMU") OR EXCLUDE(SUBJAREA,"BIOC") OR EXCLUDE(SUBJAREA,"NEUR") OR EXCLUDE(SUBJAREA,"PHAR") OR EXCLUDE(SUBJAREA,"AGRI") OR EXCLUDE(SUBJAREA,"IMMU") OR EXCLUDE(SUBJAREA,"ENVI") OR EXCLUDE(SUBJAREA,"ECON") OR EXCLUDE(SUBJAREA,"ENGI") OR EXCLUDE(SUBJAREA,"BUSI") OR EXCLUDE(SUBJAREA,"COMP") OR EXCLUDE(SUBJAREA,"MATH") OR EXCLUDE(SUBJAREA,"DECI") OR EXCLUDE(SUBJAREA,"VETE") OR EXCLUDE(SUBJAREA,"CENG") OR EXCLUDE(SUBJAREA,"EART") OR EXCLUDE(SUBJAREA,"ENER") OR EXCLUDE(SUBJAREA,"PHYS") OR EXCLUDE(SUBJAREA,"CHEM") OR EXCLUDE(SUBJAREA,"MATE") OR EXCLUDE(SUBJAREA,"BIOC") OR EXCLUDE(SUBJAREA,"NEUR") OR EXCLUDE(SUBJAREA,"PHAR") OR EXCLUDE(SUBJAREA,"AGRI") OR EXCLUDE(SUBJAREA,"IMMU") OR EXCLUDE(SUBJAREA,"ENVI") OR EXCLUDE(SUBJAREA,"ECON") OR EXCLUDE(SUBJAREA,"ENGI") OR EXCLUDE(SUBJAREA,"BUSI") OR EXCLUDE(SUBJAREA,"COMP") OR EXCLUDE(SUBJAREA,"MATH") OR EXCLUDE(SUBJAREA,"DECI") OR EXCLUDE(SUBJAREA,"VETE") OR EXCLUDE(SUBJAREA,"CENG") OR EXCLUDE(SUBJAREA,"EART") OR EXCLUDE(SUBJAREA,"ENER") OR EXCLUDE(SUBJAREA,"PHYS") OR EXCLUDE(SUBJAREA,"CHEM") OR EXCLUDE(SUBJAREA,"MATE")) AND (LIMIT-TO(DOCTYPE,"ar")) AND (LIMIT-TO(LANGUAGE,"English")) AND (LIMIT-TO(SRCTYPE,"j")))

SCOPUS search term to track trends (substitute xyz for terms listed in paper):

((TITLE(intervention) OR TITLE(trial) AND TITLE-ABS-KEY(health w/5 behav*) AND NOT TITLE (meta-analy*) AND NOT TITLE (meta analy*) AND NOT TITLE(review)) AND ALL (xyz) AND SUBJAREA(MULT OR MEDI OR NURS OR VETE OR DENT OR HEAL OR MULT OR ARTS OR BUSI OR DECI OR ECON OR PSYC OR SOCI) AND PUBYEAR > 1979 AND PUBYEAR < 2014 AND (EXCLUDE(SUBJAREA,"BIOC") OR EXCLUDE(SUBJAREA,"NEUR") OR EXCLUDE(SUBJAREA,"PHAR") OR EXCLUDE(SUBJAREA,"AGRI") OR EXCLUDE(SUBJAREA,"IMMU") OR EXCLUDE(SUBJAREA,"BIOC") OR EXCLUDE(SUBJAREA,"NEUR") OR EXCLUDE(SUBJAREA,"PHAR") OR EXCLUDE(SUBJAREA,"AGRI") OR EXCLUDE(SUBJAREA,"IMMU") OR EXCLUDE(SUBJAREA,"ENVI") OR EXCLUDE(SUBJAREA,"ECON") OR EXCLUDE(SUBJAREA,"ENGI") OR EXCLUDE(SUBJAREA,"BUSI") OR EXCLUDE(SUBJAREA,"COMP") OR EXCLUDE(SUBJAREA,"MATH") OR EXCLUDE(SUBJAREA,"DECI") OR EXCLUDE(SUBJAREA,"VETE") OR EXCLUDE(SUBJAREA,"CENG") OR EXCLUDE(SUBJAREA,"EART") OR EXCLUDE(SUBJAREA,"ENER") OR EXCLUDE(SUBJAREA,"PHYS") OR EXCLUDE(SUBJAREA,"CHEM") OR EXCLUDE(SUBJAREA,"MATE") OR EXCLUDE(SUBJAREA,"BIOC") OR EXCLUDE(SUBJAREA,"NEUR") OR EXCLUDE(SUBJAREA,"PHAR") OR EXCLUDE(SUBJAREA,"AGRI") OR

EXCLUDE(SUBJAREA,"IMMU") OR EXCLUDE(SUBJAREA,"ENVI") OR EXCLUDE(SUBJAREA,"ECON") OR EXCLUDE(SUBJAREA,"ENGI") OR EXCLUDE(SUBJAREA,"BUSI") OR EXCLUDE(SUBJAREA,"COMP") OR EXCLUDE(SUBJAREA,"MATH") OR EXCLUDE(SUBJAREA,"DECI") OR EXCLUDE(SUBJAREA,"VETE") OR EXCLUDE(SUBJAREA,"CENG") OR EXCLUDE(SUBJAREA,"EART") OR EXCLUDE(SUBJAREA,"ENER") OR EXCLUDE(SUBJAREA,"PHYS") OR EXCLUDE(SUBJAREA,"CHEM") OR EXCLUDE(SUBJAREA,"MATE")) AND (LIMIT-TO(DOCTYPE,"ar")) AND (LIMIT-TO(LANGUAGE,"English")) AND (LIMIT-TO(SRCTYPE,"j")))

SCOPUS search terms:

inequality*
income
gender
ethnic*
"social support"
socio-economic OR socioeconomic
norms
poverty
cultur*
"social network"
peer
"social environment"
ecologic*
multilevel OR multi-level
hierarchical
disparit*

psycholog*
sociolog*
anthropolog*
geograph*
economic*
"health economics"
epidemiolog*
