# Supplementary material for: How do health behaviour interventions take account of social context? A literature trend and co-citation analysis
Source: Health (London). 2017 Mar 23;22(4):389–410. doi: 10.1177/1363459317695630 (PMC6377061; doi:10.1177/1363459317695630)
Supplement: Supplementary material [file Web_Appendix_2.pdf]

Record No.	Author	Date	Short title
1	Bandura	1986	Social Foundations of Thought and Action
2	Bandura	1997	Self-efficacy
3	Ajzen	1991	Theory of planned behaviour
4	Cohen	1988	Statistical power analysis
5	Bandura	1977	Self-efficacy
6	Gollwitzer	1999	Implementation intentions
7	Gollwitzer	2006	Implementation intentions and goal intentions
8	Armitage	2001	Theory of Planned Behaviour
9	Prochaska	1983	Stages and processes of self-change
10	Godin	1996	Theory of planned behaviour
11	Prochaska	1992	In search of how people change
12	Baron	1986	Moderator-mediator
13	MacKinnon	2002	A comparison of methods to test mediation
14	Michie	2004	Interventions to change health behaviours
15	Webb	2006	Does changing behavioural intentions
16	Baranowski	2000	Gimme 5 fruit, juice and veg
17	Fishbein	1975	Belief, attitude, intention and behav
18	Brug	2003	Past, present and future
19	Kroeze	2006	A systematic review of randomized trials
20	Hettema	2005	Motivational interviewing
21	Miller	2002	Motivational interviewing
22	Burke	2003	Physical activity and nutrition programs
23	Ajzen	1980	Understanding attitudes and predicting social behavior.
24	Bandura	2004	Health promotion by social cognitive
25	Oenema	2005	Short-term efficacy of a web-based
26	Gollwitzer	1993	Implementations: meta-analysis
27	Abraham	2008	A taxonomy of behaviour
28	Dunn	2001	The use of brief interventions
29	Rubak	2005	Motivational interviewing
30	Rollnick	2008	Motivational interviewing
31	Sniehotta	2005	Action planning and coping planning
32	Schwarzer	2008	Modeling health behaviour change
33	Janz	1984	The health belief model
34	Noar	2007	Does tailoring matter
35	Miller	1991	Motivational interviewing
36	Marcus	1992	Using the stages of change model
37	Sallis	1987	Development of scales to measure
38	Cohen	1992	A power primer
39	de Vries	1999	Computer-tailored interventions
40	Goldstein	2004	Multiple behavioural risk factor
41	Whitlock	2002	Evaluating primary care
42	Radloff	1977	The CES D scale
43	Cohen	1983	A global measure of perceived stress
44	Sobell	1992	Timeline follow back
45	Ajzen	1985	From intentions to actiosn
46	Prochaska	1997	The transtheoretical model of health
47	Cole	2000	Establishing a standard definition
48	Preacher	2008	Asymptotic and resampling strategies
49	Craig	2008	Developing and evaluation complex interventions

50	Vandelanotte	2007	Website delivered physical activity
51	Cook	1979	Quasi-experimentation
52	Fisher	1992	Changing AIDS-risk behavior
53	Robinsno	1999	Reducing children's television viewing to prevent obesity
54	Summerbell	2005	Interventions for preventing
55	Glasgow	1999	Evaluating the public health
56	Rogers	2003	Diffusion of innovations
57	Liang	1986	Longitudinal data analysis
58	Bien	1993	Brief interventions for alcohol
59	Ogden	2006	Prevalence of overweight and obesity
60	Moyer	2002	Brief interventions for alcohol problems
61	Ammerman	2002	The efficacy of behavioral interventions
62	Ware	1992	The MOS 36-item short-form health survey
63	Zigmond	1983	The hospital anxiety and depression scale
64	Murray	1998	Design and Analysis of Group-Randomized Trials
65	Sallis	2000	Assessment of physical activity by self-report
66	Rubin	1987	Multiple Imputation for Nonresponse in Surveys
67	Schafer	2002	Missing data
68	Eakin	2007	Telephone interventions for physical activity and dietary b
69	Bandura	2001	Social cognitive theory
70	Raudenbush	2002	Hierarchical Linear Models
71	Godin	1985	A simple method to assess exercise behavior
72	Lobstein	2004	Obesity in children and young people
73	Deci	1985	Intrinsic Motivation and Self-Determination
74	Deci	2000	The "What" and "Why" of Goal Pursuits
75	Bandura	1989	Perceived self efficacy in the exercise
76	Lazarus	1984	Stress, appraisal and coping
77	Israel	1998	Review of community-based research
78	Saunders	1993	Development of the alcohol Use
79	Donner	2000	Design and Analysis of Cluster Randomization
80	Glasgow	2003	Why don't we see more translation
81	Eysenbach	2005	The law of attrition
82	Campbell	2004	CONSORT statement
83	Knowler	2002	Reduction in the incidence of type 2 diabetes with
84	Pate	1995	Physical activity and public health
85	Mokdad	2004	Actual causes of death in the United States
86	Webb	2010	Using the internet to promote health behaviour change
87	Folstein	1975	"mini-Mental State"
88	Beck	1996	Manual for the Beck Depression Inventory-II
89	Rosenberg	1965	Society and adolescent self image
90	Bush	1998	The AUDIT alcohol consumption
91	Morisky	1986	Concurrent and predictive validity of a self-reported meas
92	Craig	2003	International physical activity questionnaire
93	Hollis	1999	What is meant by intention to treat analysis?
94	Schulz	2010	CONSORT 2010 Statement
95	Goodman	1997	The Strengths and Difficulties Questionnaire
96	Lovibond	1995	Manual for the depression anxiety stress scales
97	Tate	2001	Using Internet technology to deliver
98	Braun	2006	Using thematic analysis in psychology
99	Vickers	2001	Analysing controlled trials

100	Achenbach	1991	Integrative Guide for the 1991

AR coding	DH coding	Difference	Decision	Final coding	
0	0	0		0	1= Social context pape
0	0	0		0	
0	0	0		0	
0	0	0		0	
0	0	0		0	
0	0	0		0	
0	0	0		0	
0	0	0		0	
0	0	0		0	
0	0	0		0	
0	0	0		0	
0	0	0		0	
0	0	0		0	
0	0	0		0	
0	0	0		0	
0	0	0		0	
0	0	0		0	
1	1	0		1	
1	1	0		1	
0	0	0		0	
0	0	0		0	
0	0	0		0	
0	0	0		0	
1	0	-1	1	1	
0	1	1	0	0	
0	0	0		0	
0	0	0		0	
0	0	0		0	
0	0	0		0	
0	0	0		0	
0	0	0		0	
0	0	0		0	
0	0	0		0	
1	1	0		1	
0	0	0		0	
0	0	0		0	
1	1	0		1	
0	0	0		0	
1	1	0		1	
0	0	0		0	
0	0	0		0	
0	0	0		0	
0	0	0		0	
0	0	0		0	
0	0	0		0	
0	0	0		0	
0	0	0		0	
0	0	0		0	
1	0	-1	0	0	

0	1	1	0	0
0	0	0		0
0	0	0		0
0	0	0		0
1	0	-1	1	1
1	0	-1	1	1
1	1	0		1
0	0	0		0
0	0	0		0
1	0	-1	0	0
0	0	0		0
0	0	0		0
0	0	0		0
0	0	0		0
0	0	0		0
0	0	0		0
0	0	0		0
0	0	0		0
0	0	0		0
0	0	0		0
0	0	0		0
0	1	1	0	0
0	0	0		0
0	0	0		0
1	0	-1	1	1
0	0	0		0
0	0	0		0
1	1	0		1
0	0	0		0
0	0	0		0
0	1	1	1	1
0	0	0		0
0	0	0		0
0	0	0		0
0	0	0		0
0	0	0		0
0	0	0		0
0	0	0		0
0	0	0		0
1	0	-1	1	1
0	0	0		0
0	0	0		0
0	0	0		0
0	0	0		0
0	0	0		0
0	0	0		0
0	0	0		0
0	0	0		0
0	1	1	1	1
0	0	0		0

0	0	0	0
			14

rs

Record no.	Author	Date	Short title
1	Ajzen	1980	Understanding Attitudes and Predicting Social Behavior
2	Bandura	1986	Social Foundations of Thought
3	Bandura	1997	Self-efficacy
4	Prochaska	1992	In search of how people change
5	Fishbein	1975	Belief, Attitude, Intention and Behavior
6	Ajzen	1991	Organizational Behavior and Human
7	Bandura	1977	Self-efficacy
8	Bandura	1994	Social cognitive theory and control
9	Fisher	1992	Changing AIDS-risk behavior
10	Miller	1991	Motivational Interviewing
11	Baron	1986	The moderator-mediator variable distinction
12	Janz	1984	The health belief model
13	Marcus	1992	The stages and processes of exercise
14	Prochaska	1983	Stages and processes of self-change
15	Becker	1974	The health belief model and personal
16	Baranowski	1998	Mediating variable framework in physical activity interventions
17	Pate	1995	Physical activity and public health
18	Sallis	1985	Physical activity assessment methodology
19	Jemmott	1992	Reductions in HIV risk-associated sexual
20	Rosenstock	1988	Social learning model and healt belief model
21	Marcus	1992	Self-efficacy and the stages of
22	Ajzen	1986	Prediction of goal-directed behavior
23	Anderson	1992	The effect of general practitioners'
24	Wallace	1988	Randomised controlled trial of general practitioner
25	Kalichman	1996	Experimental component analysis of a behavioral
26	Kalichman	1996	Prevention of sexually transmitted HIV
27	Kristal	1990	Patterns of dietary behaviour
28	Winer	1971	Statistical principles in experimental design
29	Prochaska	1992	Stages of change in the modification
30	Kelly	1995	Advances in HIV/AIDS education
31	Becker	1974	The health belief model and personal
32	Cook	1979	Quasi-Experimentation
33	Marcus	1992	Using the stages of change model to
34	Rosenstock	1974	Historical origins of the health belief model
35	Ajzen	1985	From intentions to actions
36	Blair	1985	Assessment of habitual physical activity
37	DiClemente	1995	A randomized controlled trial of an HIV
38	Kauth	1991	Reliability of retrospective assessments
39	Prochaska	1984	The Transtheoretical Approach
40	Marcus	1992	Assessing motivational readiness and decision
41	Brug	1996	The impact of a computer-tailored nutrition intervention
42	Skinner	1994	Physicians' recommendations for mammography
43	Bandura	1989	Perceived self efficacy in the exercise
44	Ajzen	1988	Attitudes, Personality, and Behavior
45	Dishman	1994	Determinants and interventions for physical activity
46	King	1991	Group- vs home-based exercise training
47	Prochaska	1986	Toward a comprehensive model of change
48	McLeroy	1988	An ecological perspective on health
49	Dishman	1996	Increasing physical activity
50	Miller	1991	Motivational Interviewing

51	Rollnick	1999	Health Behavior Change
52	Godin	1985	A simple method to assess exercise behavior
53	Godin	1996	The theory of planned behavior
54	Rogers	1983	Diffusion of innovations
55	Green	1991	Health promotion planning
56	Rogers	1983	Diffusion of innovations
57	Cohen	1988	Statistical Power Analysis for the Behavioral Sciences
58	Littell	1996	SAS System for Mixed Models
59	Murray	1998	Design and Analysis of Group-Randomized Trials
60	Bandura	1982	Self-efficacy mechanism in human agency
61	Calfas	1996	A controlled trial of physician counseling
62	Sallis	1999	Physical Activity and Behavioral Medicine
63	Amaro	1995	Love, sex and power
64	Catania	1990	Toward an understanding of risk behavior
65	Marlatt	1985	Relapse Prevention
66	Prochaska	1993	Standardized, individualized, interactive and personalized
67	Glasgow	1999	Evaluating the public health impact of health promotion interve
68	Stokols	1996	Translating social ecological theory
69	Weinstein	1988	The precaution adoption process
70	Luepker	1996	Outcomes of a field trial to improve children's
71	Radloff	1977	The CES-D scale: a self-report
72	Prochaska	1997	The transtheoretical model of health
73	Bien	1993	Brief interventions for alcohol problems
74	Murray	1994	Analysis issues in the evaluation of community trials
75	Jessor	1977	Problem Behavior and Psychosocial Development
76	Abrams	1994	Cancer control at the workplace
77	Ware	1992	The MOS 36-item short-form health survey
78	Rogers	1995	Diffusion of innovations
79	Kegeles	1996	The Mpowerment project
80	Rosenberg	1965	Society and adolescent self image
81	Donner	1981	Randomization by cluster
82	Liang	1986	Longitudinal data analysis using generalized
83	Bronfenbrenner	1979	The Ecology of Human Development
84	Jemmott	1998	Abstinence and safer sex HIV risk reduction
85	Hawkins	1992	Risk and protective factors for alcohol
86	Susser	1995	The tribulations of trials
87	Rubin	1987	Multiple Imputation for Nonresponse in Surveys
88	Fleming	1997	Brief physician advice for problem alcohol drinkers
89	Oakley	1995	Behavioural interventions for HIV/AIDS prevention
90	Rollnick	1992	Negotiating behaviour change in medical settings
91	McGinnis	1993	Actual causes of death in the United States
92	Prochaska	1983	Stages and processes of self-change of smoking
93	Cupples	1994	Randomised controlled trial of health promotion
94	Carleton	1995	The Pawtucket heart health program
95	Campbell	1994	Improving dietary behaviour
96	Oxman	1995	No magic bullets
97	Lazarus	1984	Stress, Appraisal, and Coping
98	US PSTF	1996	Guide to Clinical Preventive Services
99	APA	1994	Diagnostic and Statistical Manual of Mental Disorders
100	Achenbach	1991	Integrative Guide for the 1991

AR coding	DH coding	Difference	Decision	Final coding		
0	0	0		0		1= Social context pape
0	0	0		0		
0	0	0		0		
0	0	0		0		
0	0	0		0		
0	0	0		0		
0	0	0		0		
0	0	0		0		
0	0	0		0		
0	0	0		0		
0	0	0		0		
0	0	0		0		
0	0	0		0		
0	0	0		0		
0	0	0		0		
0	0	0		0		
0	0	0		0		
1	1	0		1		
1	1	0		1		
0	0	0		0		
0	0	0		0		
0	0	0		0		
0	0	0		0		
0	0	0		0		
0	1	1	1	1		
0	0	0		0		
0	0	0		0		
0	0	0		0		
0	0	0		0		
1	0	-1	1	1		
0	0	0		0		
0	0	0		0		
0	0	0		0		
0	0	0		0		
0	0	0		0		
0	1	1	0	0		
1	1	0		1		
0	0	0		0		
0	0	0		0		
0	1	1	0	0		
1	1	0		1		
1	1	0		1		
0	0	0		0		
0	0	0		0		
0	1	1	1	1		
1	1	0		1		
0	0	0		0		
1	1	0		1		
1	0	-1	1	1		
0	0	0		0		

0	0	0		0
0	1	1	0	0
0	0	0		0
1	0	-1	1	1
0	1	1	1	1
1	1	0		1
0	0	0		0
0	0	0		0
1	0	-1	0	0
0	0	0		0
0	0	0		0
0	0	0		0
1	1	0		1
0	0	0		0
0	0	0		0
0	1	1	0	0
1	1	0		1
1	1	0		1
0	0	0		0
1	1	0		1
0	0	0		0
0	0	0		0
0	0	0		0
0	0	0		0
1	1	0		1
1	0	-1	1	1
0	0	0		0
1	1	0		1
1	1	0		1
1	1	0		1
1	0	-1	1	1
0	0	0		0
1	1	0		1
1	1	0		1
1	0	-1	1	1
1	1	0		1
0	0	0		0
1	1	0		1
0	0	0		0
0	0	0		0
1	0	-1	0	0
0	0	0		0
0	1	1	0	0
1	1	0		1
1	1	0		1
0	0	0		0
0	0	0		0
0	0	0		0
0	0	0		0
0	0	0		0

rs
